# DNA Copy Number Aberrations, and Human Papillomavirus Status in Penile Carcinoma. Clinico-Pathological Correlations and Potential Driver Genes

**DOI:** 10.1371/journal.pone.0146740

**Published:** 2016-02-22

**Authors:** Susannah La-Touche, Christophe Lemetre, Maryou Lambros, Elzbieta Stankiewicz, Charlotte K. Y. Ng, Britta Weigelt, Ramzi Rajab, Brendan Tinwell, Cathy Corbishley, Nick Watkin, Dan Berney, Jorge S. Reis-Filho

**Affiliations:** 1 Bart's Cancer Institute, Centre for Molecular Oncology, Queen Mary University of London, John Vane Science Centre, Charterhouse square, London, United Kingdom; 2 Department of Pathology, Memorial Sloan-Kettering Cancer Center, New York, New York, United States of America; 3 Molecular Pathology, Institute of Cancer Research, London, United Kingdom; 4 St George’s Hospital, Tooting, London, United Kingdom; 5 Human Oncology and Pathogenesis Program, Memorial Sloan-Kettering Cancer Center, New York, New York, United States of America; Georgetown University, UNITED STATES

## Abstract

Penile squamous cell carcinoma is a rare disease, in which somatic genetic aberrations have yet to be characterized. We hypothesized that gene copy aberrations might correlate with human papillomavirus status and clinico-pathological features. We sought to determine the spectrum of gene copy number aberrations in a large series of PSCCs and to define their correlations with human papillomavirus, histopathological subtype, and tumor grade, stage and lymph node status. Seventy formalin-fixed, paraffin embedded penile squamous cell carcinomas were centrally reviewed by expert uropathologists. DNA was extracted from micro-dissected samples, subjected to PCR-based human papillomavirus assessment and genotyping (INNO-LiPA human papillomavirus Genotyping *Extra* Assay) and microarray-based comparative genomic hybridization using a 32K Bacterial Artificial Chromosome array platform. Sixty-four samples yielded interpretable results. Recurrent gains were observed in chromosomes 1p13.3-q44 (88%), 3p12.3-q29 (86%), 5p15.33-p11 (67%) and 8p12-q24.3 (84%). Amplifications of 5p15.33-p11 and 11p14.1-p12 were found in seven (11%) and four (6%) cases, respectively. Losses were observed in chromosomes 2q33-q37.3 (86%), 3p26.3-q11.1 (83%) and 11q12.2-q25 (81%). Although many losses and gains were similar throughout the cohort, there were small significant differences observed at specific loci, between human papillomavirus positive and negative tumors, between tumor types, and tumor grade and nodal status. These results demonstrate that despite the diversity of genetic aberrations in penile squamous cell carcinomas, there are significant correlations between the clinico-pathological data and the genetic changes that may play a role in disease natural history and progression and highlight potential driver genes, which may feature in molecular pathways for existing therapeutic agents.

## Introduction

Penile squamous cell carcinoma (PSCC) is a rare disease, but can have a profound physical and psychological effect on those afflicted. It mainly affects men aged 50–70 years old [1] and, although rare in developed countries, it is a significant health problem in developing countries. In Europe and the USA the incidence is less than 1 per 100,000 men, but the age-standardized rate may be as high as 3.7 and 2.8 per 100,000 in parts of Brazil and Uganda, respectively [2–4]. Penile carcinoma is difficult to treat after metastatic dissemination, given that these tumors are relatively radio and chemo resistant.

Histologically, PSSCs comprise a spectrum of lesions that differ in clinical behavior. The most common morphological subtypes of PSCC are usual type (48–65%), basaloid (4–10%), warty (7–10%) and verrucous (3–8%) [5]. Basaloid carcinomas are the most aggressive PSCC subtype, whereas verrucous carcinomas have a more indolent clinical course [6].

In comparison to other squamous cell carcinomas, relatively little is known about the molecular features of PSCC [7–11]. Similar to squamous cell carcinomas of the female lower genital tract, a subset of PSCCs appears to be driven by HPV. In a recent study analyzing 102 cases [9], HPV DNA was detected in up to 56% of these tumors; however, frequencies ranging from 14% to 100% have been reported in the literature [12]. The prevalence of HPV infection seems to correlate with histological type, with HPV DNA reported to be found in up to 76% of basaloid carcinomas and in only 22% of the indolent verrucous carcinomas [6]. Esophageal squamous cell carcinomas (OSCCs) also comprise HPV-positive and HPV-negative tumor types, with the frequency of HPV DNA detection being highly variable in both [6, 9, 13–15], which may reflect the discrepancies in detection methodology [16]. In oropharyngeal squamous cell carcinoma (OPSCC) HPV positivity confers a good prognosis [17] and better disease free survival [18] compared to HPV-negative cases. HPV infection has also been well documented in other squamous cell carcinomas such as vulva and cervical.

There is a paucity of data on the somatic genetic characteristics of PSSCs, possibly due to the relative rarity of the disease. In England, PSCC services have been managed in supra-specialized centers in the UK. This has given us the opportunity to accrue a sufficiently sized cohort to analyze the molecular characteristics of PSCCs. Therefore, we sought to investigate the repertoire of somatic DNA copy number aberrations in PSSCs, and to determine whether patterns and types of gene copy number aberrations would correlate with HPV status and clinico-pathological features, including type, grade, stage and HPV status.

## Materials and Methods

### Cases

We retrieved 70 formalin fixed paraffin embedded PSCCs from the Cellular Pathology Department of St George’s Hospital. Cases were reviewed by a panel of expert uropathologists (CC, BT, RR) for grade, stage (including lymph node status) and subtype using established methods [19, 20]. Patients’ data were made entirely anonymous and this study was approved by East London and The City Research Ethics Committee Alpha (The Orchid Tissue Bank; 09/H0704/4).

### Dissection and DNA Extraction

For all cases, regions of PSCC and normal tissue were marked on the representative sections by a consultant genitourinary pathologist (DB). Ten to fifteen tissue representative sections were cut at 10μm thickness from each case and subjected to microdissection under a stereomicroscope as previously described [21].

DNA was extracted from microdissected sections using standard phenol-chloroform methods and its concentration was measured using PicoGreen^®^ assay as per the manufacturer’s instructions (Applied Biosystems, Invitrogen, Paisley, UK).

### HPV Analysis

The presence of HPV DNA was detected by PCR method using SPF10 primers, which amplify a 65-bp fragment of the conserved L1 open reading frame and HPV genotypes identified by the INNO-LiPA HPV Genotyping *Extra* Assay (Innogenetics NV Ghent, Belgium) as previously described [9].

### Microarray Comparative Genomic Hybridization (aCGH)

DNA extracted from microdissected sections was subjected to microarray-based comparative genomic hybridization using a 32K bacterial artificial chromosome (BAC) array platform (Hernandez *et al*., 2012) which has been shown to be as robust as, and to have comparable resolution with, high-density oligonucleotide arrays [22–24]. DNA labeling, array hybridization and image acquisition were performed as previously described [25].

After filtering polymorphic BACs, a final dataset was produced with unambiguous mapping information according to build hg19 (NCBI build 37) of the human genome (http://www.ensemble.org).

### Data Analysis

Analyses were performed using the BACE.R R script (R version 2.11.1) was used to pre-process and analyze the aCGH data as previously described [25, 26].

After filtering polymorphic BACs, a final dataset of 31,367 clones with unambiguous mapping information according to build hg19 of the human genome (http://www.ensembl.org) was smoothed using the circular binary segmentation (cbs) algorithm. A categorical analysis was applied to the BACs after classifying them as representing amplification (>0.4), gain (>0.08 and ≤0.45), loss (<-0.08), or no change according to their cbs-smoothed Log_2_ ratio values [7,10,9]. Threshold values were determined and validated as previously described [21, 26].

Following copy number categorizations for gains, losses or amplifications, Fisher’s exact tests were performed to identify statistically significant differences (with a false discovery rate-adjusted p-value < 0.05) in amplifications and gains/ losses between the different subtypes, grades, stages, HPV associations and nodal status. Adjustment for multiple testing was performed with a step-down permutation procedure (maxT), which provides a robust control of the family-wise type I error rate (FWER) [27]. Unsupervised hierarchical clustering of cbs-smoothed ratios, employing Wards clustering algorithm and Euclidean distance metric, was performed as previously described [26].

Subsequent assessment of the robustness of the cluster analysis was done by assessing the uncertainty with multiscale bootstrap resampling with the pvclust package in R 3.0.1.

## Results

### Patients and Study Samples

Of the 70 PSCC cases, 64 had adequate DNA quality and purity for successful array CGH analysis.

**Fig 1:**
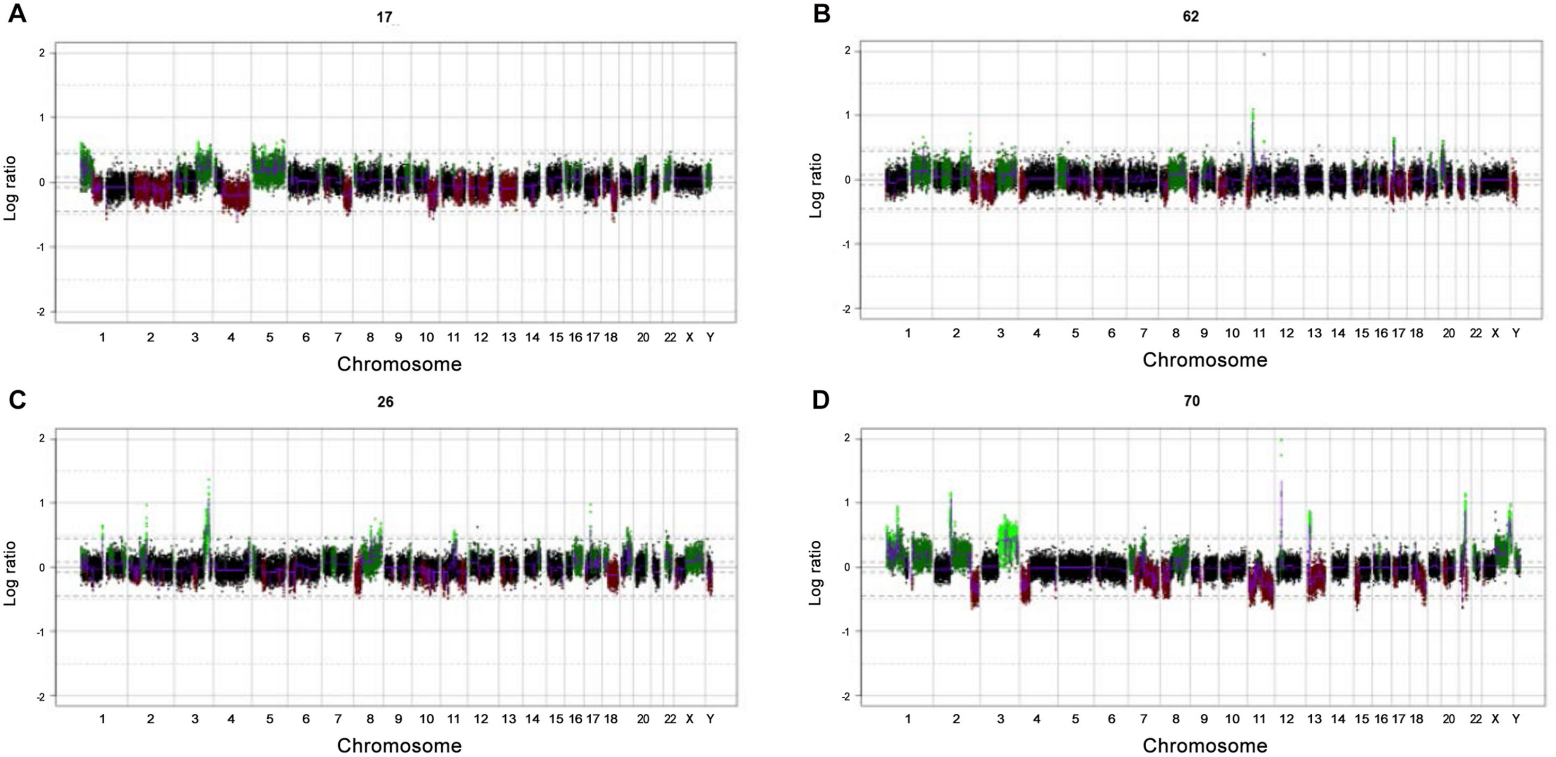
Microarray-based comparative genomic hybridisation analysis invasive penile squamous cell carcinoma components. Genome plots obtained with the 32K bacterial artificial chromosome array platform for: (A) Case 17 (usual type), (B) Case 62(verrucous), (C) Case 26 (usual type) and (D) Case 70 (basaloid).

Demographics including HPV status, stage, grade, lymph node status and histological classification can be found in Table 1. Genome plots were generated for each case and showed various patterns of chromosomal expression. Some examples are as follows: Case 17 (usual type PSCC), exhibited specific gain/amplification in regions 1p, 3q and 5 (Fig 1A). Case 62 (verrucous type PSCC) showed gains in 1q, 2, 3q, 5p and 8q, with obvious amplification in chromosome 11p (Fig 1B). Cases 26 (usual type PSCC) and 70 (basaloid type PSCC) also showed gain in the 3q region; as well as 8q and 13p, respectively (Fig 1C and 1D).

**Table 1:** Demographics of PSCC cases.

Group for Comparison	Subgroup for Comparison	Number of Cases
**Cancer Type**	Basaloid	7
	Usual	50
	Verrucous	7
**HPV status**	Positive	35
	Negative	29
**Stage**	Stage 1	4
	Stage 2	8
	Stage 2A	28
	Stage 2B	4
	Stage 3	7
	Stage 3A	8
	Stage 4	5
**Grade**	Grade 1	6
	Grade 2	17
	Grade 3	41
**Nodal Status**	Nodal N0	31
	Nodal N1	16
	Nodal N2	13
	Nodal N3	2
	No information	2

### Array CGH and Candidate Driver Genes

**Fig 2:**
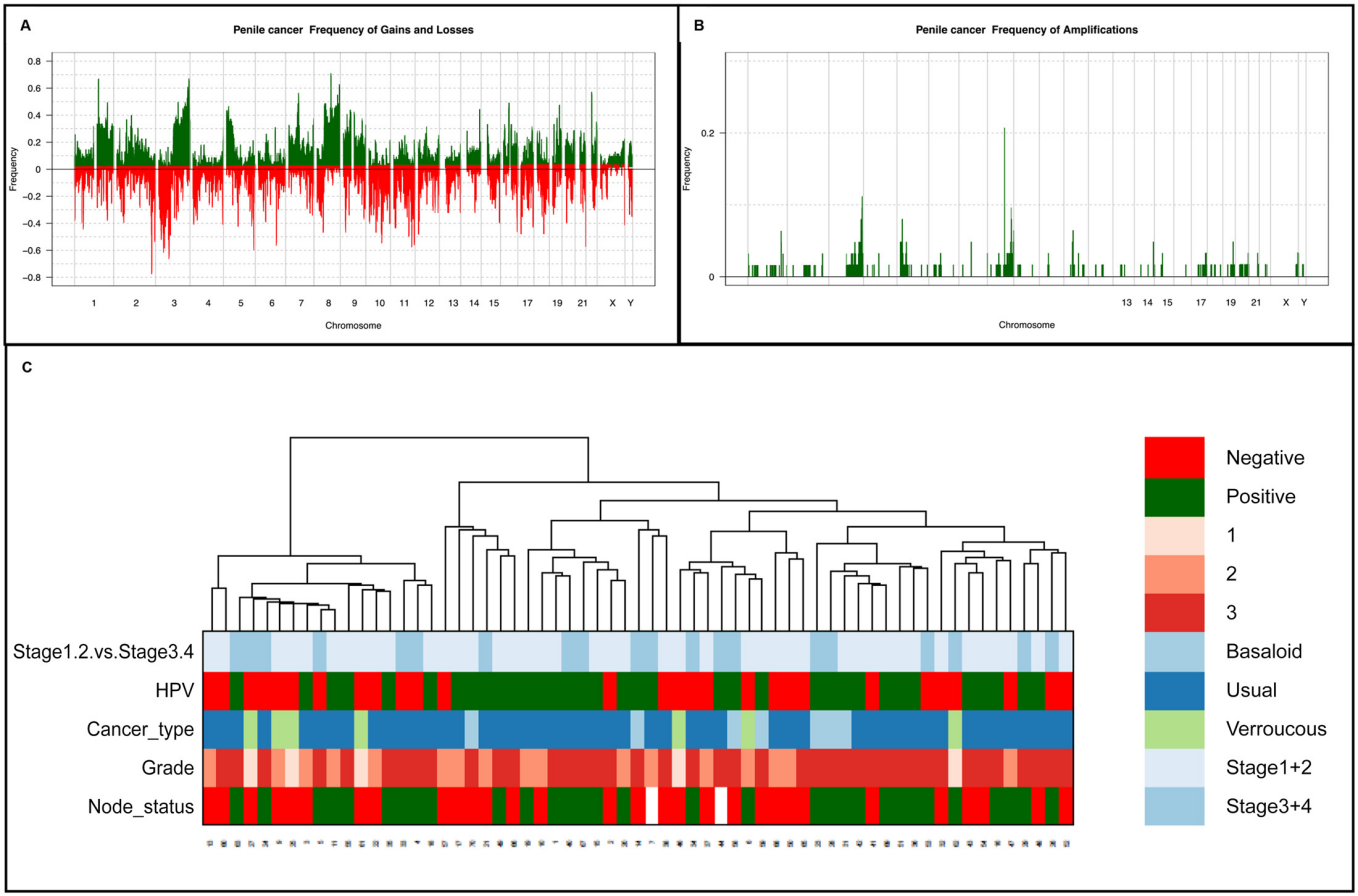
Samples of invasive penile squamous cell carcinoma. (A) Frequency plot of copy number gains and losses. (B) Frequency plot of high level gain or amplification. (C) Hierarchical cluster analysis for all cases comparing stage, HPV status, subtype, grade and nodal status. The proportion of tumors in which each bacterial artificial chromosome (BAC) clone is gained (green bars) or lost (red bars) is plotted (*y* axis) for each BAC clone according to its genomic position (*x* axis).

PSCCs were shown to harbor many highly recurrent gains, the highest frequencies occurring on chromosomes 1p13.3-q44 (88%), 3p12.3-q29 (86%), 7p22.3-q36.3 (88%), 8p12-q24.3 (84%), 9p24.3-q34.3 (88%) and 20p13-q13.33 (84%) (Fig 2A, S1 Table). PSCCs also harbored highly recurrent losses of 2q33-q37.3 (86%), 3p26.3-q11.1 (83%) and 11q12.2-q25 (81%) (Fig 2A, S1 Table). The highest frequency recurrent amplifications were of 3q27.2-q29 (11%) and 8q21.13-q24.3 (23%) (Fig 2B, S1 Table). Our analysis of PSCC samples did not identify deletions or high-level loss of genetic loci and, despite the highly recurrent changes, there were genetic aberrations that were restricted to only a subset of the cases.

These loci enclose several genes identified in areas of gain, amplification and loss. Gain of 3p12.3-q29 harbor, amongst many other genes, PRKCI, PIK3CA, DCUN1D1, LAMP3 and RPL35A. The F-box protein, SKP2 (S-phase kinase-associated protein 2) was included in gain of 5p15.33-p11 and amplification of 5p15.33-p11 and the oncogene MYC is also highlighted in the gained region 8p12-q24.3. Amplification of 3q27.2-q29 encompasses the genes PIK3CA, SENP5 (SUMO1/Sentrin Specific Peptidase 5), CLDN1, TNK2 (tyrosine kinase, non-receptor, 2) and FBXO45 (F-box protein 45). Genes of interest in the amplified region 3q26.1-q27.2 include FNDC3B and ACTL6A/BAF53 (actin-like 6A/BRG1 associated factor 53A). CPT1A, FGF3/4 and CCND1 are revealed in the amplified region 11p12.-p14.1.

Potential tumor suppressor genes, also found in other squamous cell carcinomas, have been discovered in the lost regions 2q33-q37.3 (PLCD4), 3p26.3-q11.1 (RBMS3, PLCD1, and CACNA2D3) and 11q12.2-q25 (CPT1A, CCND1, FGF4/FGF3, and PPP2R1B).

### HPV Subgroup-Analysis

Firstly, we sought to define whether HPV DNA positive and HPV DNA negative PSSCs would be underpinned by distinct patterns of gene copy number aberrations. Multi-Fisher's exact test adjusted for multiple comparisons revealed that HPV-positive and HPV-negative PSCCs have similar patterns of copy gains and that higher frequencies were demonstrated in 1q, 3q, 5p, 7p and 8q and amplifications in 3q, 5p and 8q for both HPV-negative and HPV-positive tumors.

**Fig 3:**
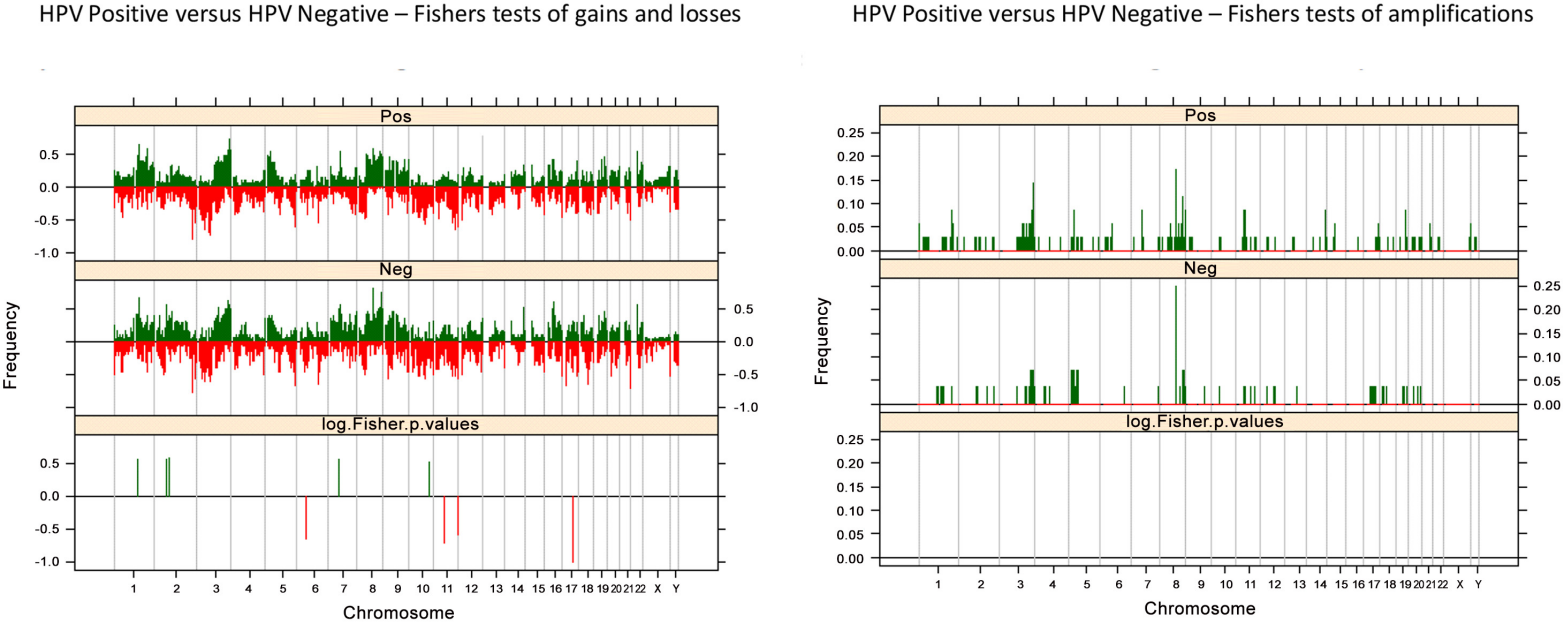
HPV status of PSCCs. Fishers Exact plots of differential copy number gains and losses or amplifications/ high level gain in HPV positive and HPV negative PSCC samples. The proportion of tumors in which each bacterial artificial chromosome (BAC) clone is gained (green bars) or lost (red bars) is plotted (*y* axis) for each BAC clone according to its genomic position (*x* axis).

Regions of differential gain included 1q, 2p, 7p and 10q, and regions of differential loss on 6p, 11p and q and 17p and q. When an unadjusted p value was used, 17p loss was exhibited with an increased frequency in the HPV negative group (p<0.05), but other areas of differential gain and loss showed no pattern of association with either the HPV positive or negative group. When p values were adjusted, regions with a statistically significant higher frequency of cases in HPV positive cohort exhibited gain on 1q (151,622,431–151,793,285; p = 0.035). When p values were adjusted, regions with a statistically significant higher frequency of cases in HPV negative cohort exhibited gain of 2p (60,835,599–61,089,333; p = 0.034), 7p (48,668,454–49,846,998; 0.034), 10q (108,751,067–109,429,862; p = 0.047) and loss of 11p (46,103,041–47,520,939; p<0.05). No significant differences in the prevalence of specific amplified loci were identified. Hierarchical cluster analysis failed to demonstrate a clear difference in clustering of genetic change between HPV-positive and HPV-negative tumors (Figs 2C and 3).

### Further Clinico-Pathological Subgroup-Analysis

**Fig 4:**
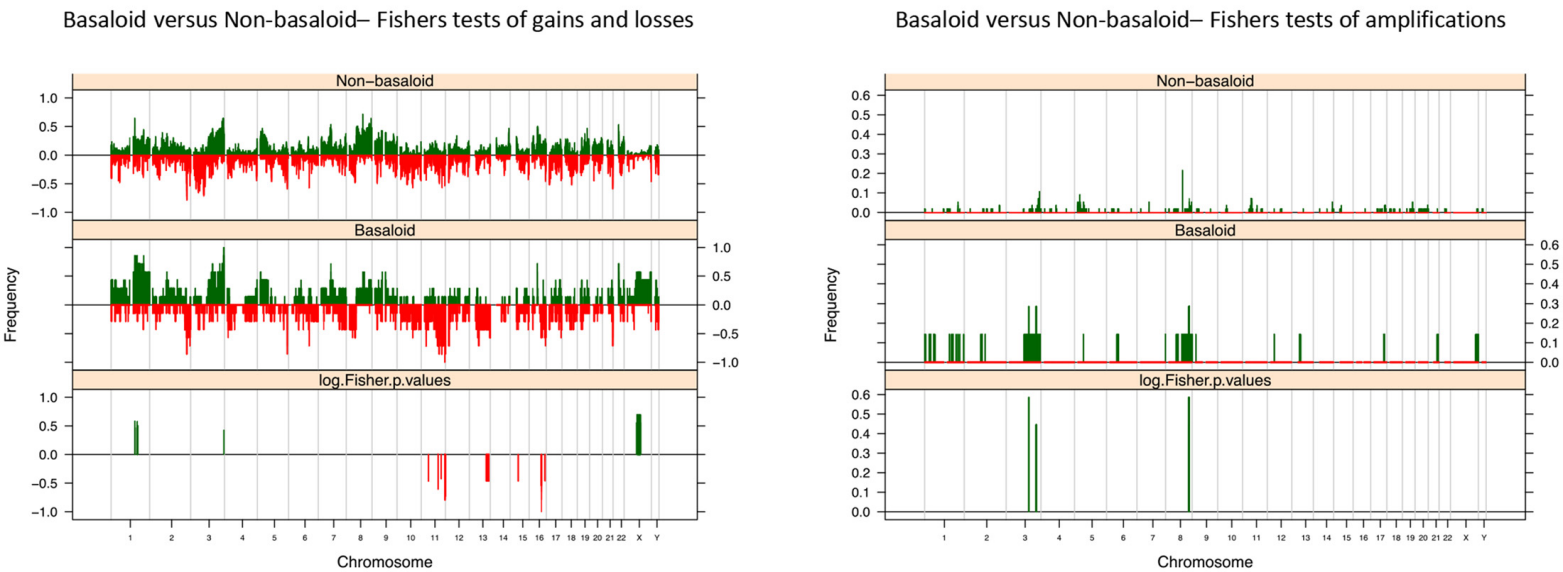
Histological subtype of PSCCs. Fishers Exact plots of differential copy number gains and losses or amplifications/ high level gain in Basaloid and non-basaloid PSCC samples. The proportion of tumors in which each bacterial artificial chromosome (BAC) clone is gained (green bars) or lost (red bars) is plotted (*y* axis) for each BAC clone according to its genomic position (*x* axis).

In addition, we conducted further analysis based on other defined clinic-pathological variables (for details of specific gene loci, see S2 Table). Analysis of histological subtypes uncovered differential gain of regions on 1q (p<0.05) and 3q(p = 0.04, region close to telomere), differential loss of 11p and q (p<0.05, close to telomere), 13q (p = 0.03), 15q (p = 0.03), 16q (p = 0.02), and differential amplification of 3q and 8q (p<0.05), when comparing basaloid to non-basaloid subtypes (Fig 4). In regions of these chromosomes statistically significant gain, loss and amplification were shown in more cases in the non-basaloid group compared to the basaloid group. Comparing verrucous cases to non-verrucous differential loss was discovered on 11p and 17q (p<0.05), in significantly more cases in the non-verrucous than verrucous cohort. No significant differences in the prevalence of specific gained or amplified loci were identified. Comparing usual type to non-usual type, differential loss was found on 3p (p<0.05) and 17q (unadjusted p<0.05, adjusted p = 0.1–0.33). Interestingly there were no significant differences in the prevalence of specific gained or amplified loci were identified.

**Fig 5:**
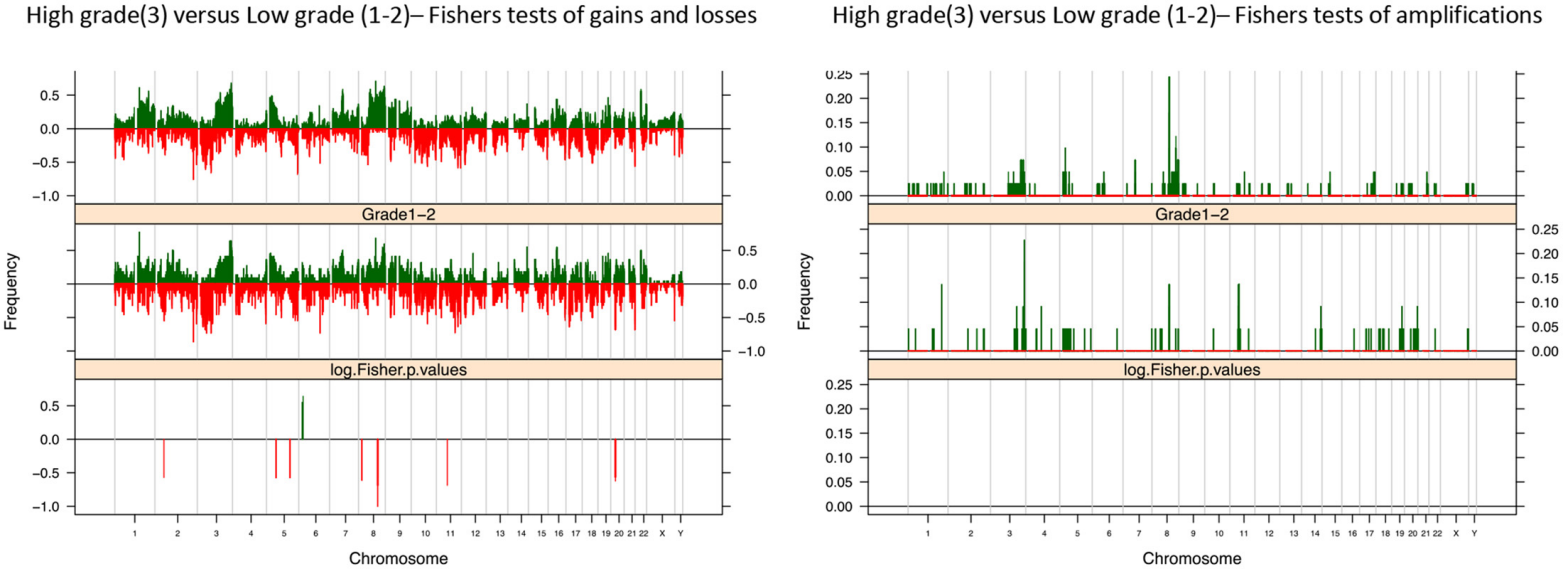
Histological grade of PSCCs. Fishers Exact plots of differential copy number gains and losses or amplifications/ high level gain in high grade (3) and low grade (1–2) PSCC samples. The proportion of tumors in which each bacterial artificial chromosome (BAC) clone is gained (green bars) or lost (red bars) is plotted (*y* axis) for each BAC clone according to its genomic position (*x* axis).

A supervised analysis of the aCGH results according to histological grade revealed differential gains in chromosome 6p and differential losses in chromosomes 2p, 5p and q, 8q and 11p. Gains of 6p were more frequently found in tumors of a low histological grade (i.e. 1 and 2) than in high-grade lesions (i.e. 3), but this was not statistically significant when adjusted p values were calculated. Loss of 2p and 5p and q were significantly more frequently found in low-grade lesions (p<0.05). Loss of 8q and 11p were significantly more frequently found in high-grade tumors (p<0.05). No significant differences in the prevalence of specific amplified loci were identified (Fig 5).

**Fig 6:**
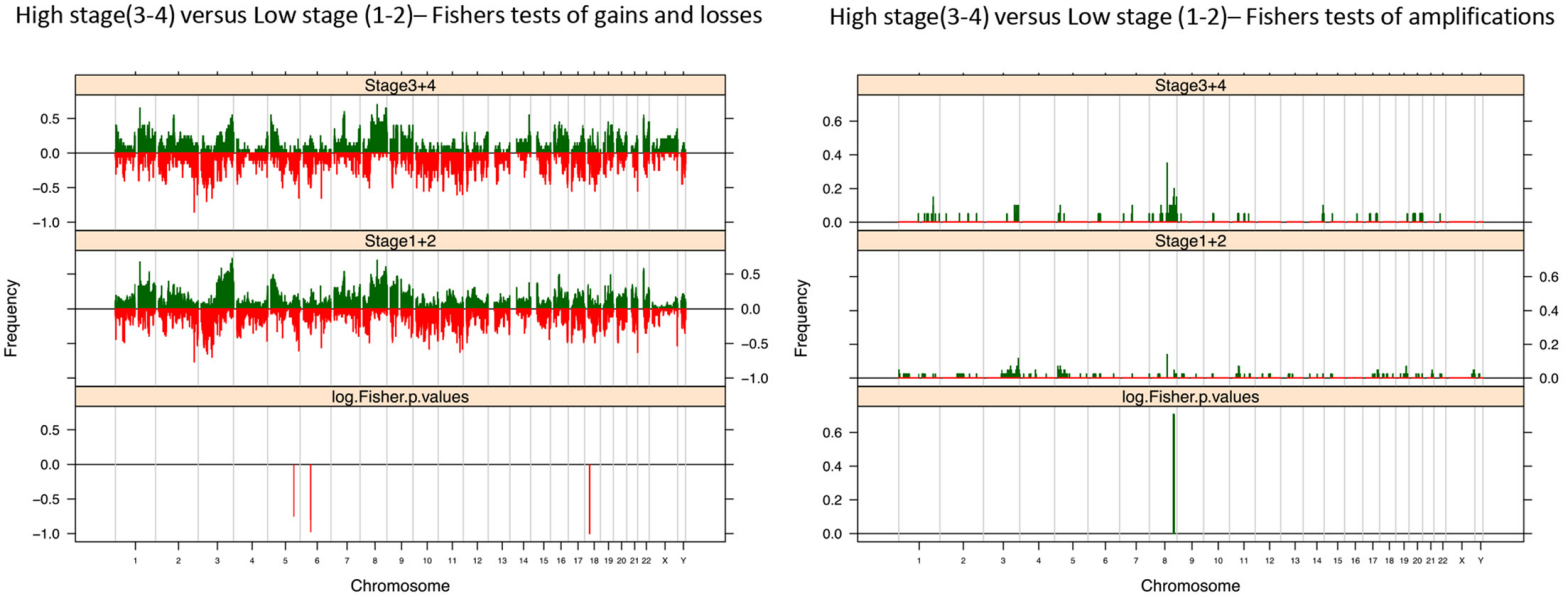
Clinical Stage of PSCCs. Fishers Exact plots of differential copy number gains and losses or amplifications/ high level gain in high stage (3–4) and low stage (1–2) PSCC samples. The proportion of tumors in which each bacterial artificial chromosome (BAC) clone is gained (green bars) or lost (red bars) is plotted (*y* axis) for each BAC clone according to its genomic position (*x* axis).

PSSCs of different stages displayed distinct patterns of differential loss on 5q (p = 0.03), 6p (p<0.01) and 18p, as well as amplification of 8q (Fig 6). These aberrations were significantly more frequently found in tumors of high histological stage (i.e. 3 and 4) than in low stage lesions (i.e. 1 and 2). No significant differences in the prevalence of specific gained loci were identified.

**Fig 7:**
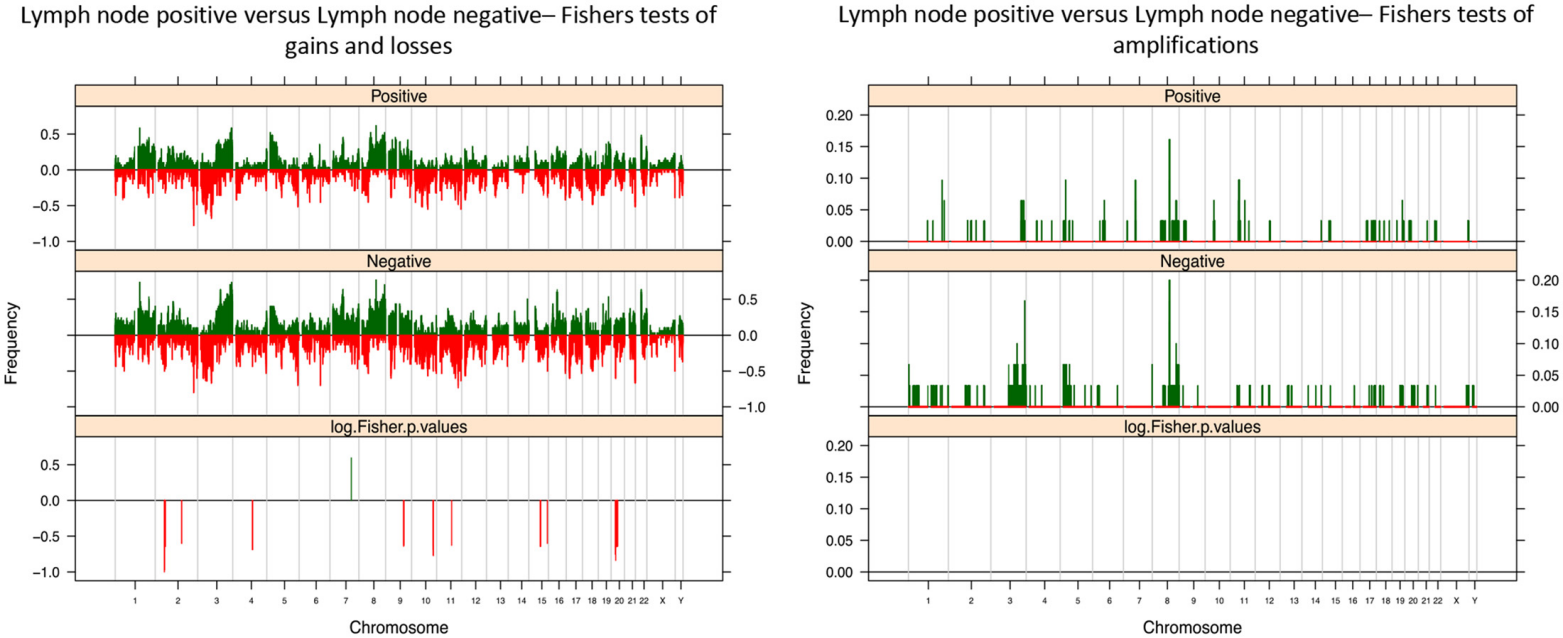
Lymph node status of PSCCs. Fishers Exact plots of differential copy number gains and losses or amplifications/ high level gain in lymph node positive and lymph node negative PSCC samples. The proportion of tumors in which each bacterial artificial chromosome (BAC) clone is gained (green bars) or lost (red bars) is plotted (*y* axis) for each BAC clone according to its genomic position (*x* axis).

Analysis of aCGH data according to nodal status (N0, N1, N2, and N3) yielded more frequent gain of 7q and loss of 2pq (p<0.05), 4q (p = 0.03), 9q (p<0.05), 10q (p<0.05), 11q (p<0.05), 15q (p<0.05) and 20p (p<0.05) in node negative cases compared to node positive cases (Fig 7). For 7q this was not statistically significant when an adjusted p value was calculated (unadjusted p = 0.001–0.04; adjusted p = 0.24–0.6). No significant differences in the prevalence of specific amplified loci were identified.

Given the diversity of gene copy number aberrations of PSSCs, we performed class discovery analysis using hierarchical clustering. There were stable clusters, but none showed enrichment for a particular clinico-pathological feature (Fig 2C).

## Discussion

We have provided a detailed characterization of the genetic landscape of prostate cancer by DNA copy number aberrations found in HPV-positive and HPV-negative PSCCs, and demonstrated that these tumors display similar patterns of genetic aberrations despite the distinct pathogenetic mechanisms, with statistically significant changes in only a few small loci. It has been documented that HPV changes cell physiology inactivating RB and p53 pathways [28, 29] and our genomic data shows copy number loss at 13q12.11-q34 (80% of HPV positive cases) and 17p13.3-p11.1 (63% of HPV positive cases), harboring RB1 and TP53 genes, respectively. However, these changes were not statistically significant in their difference from the HPV negative cohort. It is possible that TP53 and RB1 may be mutated in the cases without HPV infection. Further investigation, such as mutational analysis, will be required to elucidate this.

Another mechanism of TP53 pathway inactivation includes amplification/overexpression of an ubiquitin ligase, MDM2 (murine double minute 2). TP53 is usually kept at low levels by MDM2 in normal cells. MDM2 and TP53 form an auto-regulatory negative-feedback loop, in which TP53 induces the expression of MDM2, which then facilitates the breakdown of TP53 in the nucleus and cytoplasm, inhibits its transcriptional activity and assists its nuclear export [30]. Our data did not support this, as we failed to find any differential gain or differential amplification of this gene at 12q15 in the HPV cohorts, or amplification in the overall data. However, an earlier small genomic study confirmed amplification on 12q, but only in 4.3% of PSCC cases, which encompasses the MDM2 gene [7]. pRB1 pathway inactivation could also be caused by gains/ amplification of CCNE1, CCND1, CDK2 and CDK4, but our data did not show differential copy number change at the corresponding loci between the HPV positive and HPV negative cohorts. However, gain of the regions encompassing CCNE1 (19p13.11-q13.43; 71% cases) and CCND1 (11q12.2-q14.1; 46% cases) were found in the HPV positive group, and both gain and amplification of the region incorporating CCND1 (11q13.3-q13.4; 20% and 3% cases, respectively) were found in the HPV negative group.

One previous paper has been published on the pattern and repertoire of somatic genetic aberrations in PSSCs.[7] Alves *et al* investigated the chromosomal changes found in 26 PSCCs using a spread of metaphase chromosomes on a microscope slide as a hybridization target, prepared as in fluorescence in situ hybridization. In three cases no DNA copy number alterations were detected, but contamination of the PSCC samples with normal tissue could not be excluded. Alves *et al*. observed the most common copy number gains on 8q24 (48%), 16p11–12(48%), and 20q11–13 (48%); 22q (43%); 19q13 (39%) and 5p15 (35%). In comparison, our data demonstrated copy number gains in similar overlapping regions on chromosomes 8p12-q24.3 (84%), 16p11.2-q24.3 (77%), 20p13-q13.3 (84%), 22q11.1–13.3 (73%), 19p13.11-q13.43 (64%) and 5p15.33-q11.2 (67%). Losses were found by the Alves group on 15q, 16q, 21q and 20p. PSCC cases exhibited loss on p and q arms of all other chromosomes, excluding chromosomes 22 and Y, which did not show loss. We showed amplifications of 1q32.2 (6%), 3q27.2-q29 (11%), 3q26.1-q27.2 (8%), 5p15.2-p14.3 (9%), 5p14.3-p11 (8%), 5p15.33-p15.2 (6%), 8q21.13-q24.3 (23%) and 11p14.1-p12 (6%), none of which corresponded to the single amplified case in the Alves data set (37%). Deletions were confirmed by Alves *et al*. at 13q21–22 (43%) and 4q21–32 (30%), but our study did not detect deletions in any cases. The number of gene copy number aberrations was reported to be numerically associated with ‘shorter survival’ in Alves *et al*., but it was not made clear whether this was disease-free, disease-specific or overall survival. Our study did not assess survival data for comparison with this. Given the relatively low resolution of the methods employed by Alves et al. and lack of information on the HPV status of the tumors analyzed in their study, it is difficult to grasp the overall clinical and biological significance of their data. However, a more recent study utilizing aCGH describes copy number alterations in PSCCs and supports the hypothesis that PSCCs manifest different aetiologies according to viral status.[31]

Our current data has highlighted patterns of copy number gains, losses and amplifications in a PSCC cohort that bear some similarities to those in other HPV related squamous cell carcinomas (Table 2). For example, gains and amplifications on chromosomes 3q, 8q, 11pq and 5p, and losses on 3p and 4q are shared many squamous cell carcinomas. Gain of 3p12.3-q29 in our study harbors PIK3CA, which codes for the p110α subunit of PI3-kinase, as part of the PI3-kinase/AKT signaling pathway. It plays a key function in the regulation of cell growth and apoptosis, and it has been associated with cervical, head and neck and upper esophageal squamous cell carcinomas [32]. Also located in this region is PRKCI, a member of the protein kinase C (PKC) family of serine/threonine protein kinases involved in a wide variety of cellular processes and it has been associated with lymph node metastases in OSCC [33].

**Table 2:** Copy Number Changes in Squamous Cell Carcinomas.

Type of SCC	Author	Year	Cases/ Method	HPV data (y/n)	Gains (Candidate genes)	Losses (Candidate genes)	Amplifications (Candidate genes)	Deletions (Candidate genes)
**OSCC**	Carneiro	2008	30/ 32k aCGH	N	**5p,7pq, 8q, 10q, 11q, 12p,14q, 16p, 17p, 19pq, 20q**	**3p,5q,8p,9p,11q**	**7p***(EGFR)*, **11q** *(MYEOV*, *CCND1*, *FGF3*, *FGF4*, *PPFIA*, *FAD*, *TMEM16A*, *CTTS*, *SHANK2)*, **11q22** *(PDGF)*	9p *(CDKN2A)*
**OSCC**	Hirasaki	2007	23/ 4K aCGH	N	**3q** *(MDS1*, *LOC344887*, *ETV5*, *LOC401102/10*, *LOC440995*, *MFI2*, *LOC391609*, *DLG1*, *SKIL*, *CLDN11*, *KIAA1613*, *MCF2L2*, *B3GNT5*, *LRCH3*, *IQCG*, *KCNAB1*, *TNFSF10*, *PIK3CA*, *KCNMB3*, *AP2M1*, *ABCF3*, *LOC90113*, *ALG3*, *MGC2408*, *ECE2*, *CAM-KIIN*, *EPHB3*, *LPP*, *THPO*, *CHRD*, *LOC285248*, *MASP1*, *LOC442100*, *BDH)*, **3p** *(LOC389105*, *ARPP-21*, *FHIT*, *PTPRG*, *C3orf14)*, **11q** *(PPFIA1*, *CTTN*, *SHANK2*, *CCND1*, *FLJ42258*, *ORAOV1*, *FGF4*, *FGF3*, *CPT1A*, *OR2AL1P*, *OPCML)*, **8q** *(ZC3HDC3*, *GSDMDC1*, *PP3856*, *EEF1D)*, **4q, 5q, 18q** *(LOC400662)*	**3p** *(FHIT*, *PTPRG*, *C3orf14*, *FHIT*, *LOC391555)*, **4p, 4q** *(LOC402191)*, **5q** *(ACSL6*, *IL3*, *CSF2*, *KCNIP1)*, **11q** *(OPCML*, *NCAM1)*, **13q, 18q** *(LOC400662*, *NETO1)*	**11q**	**16p** *(ZNF434*, *ZNF174*, *ZNF597*, *FLJ14154*, *LOC390671*, *CLUAP1)*
**OSCC**	Sakai	2010	51/ CGH metaphase spread	N	n/a	n/a	**1q, 3q, 5p, 8q, 9q, 11q, 17pq, 20q, 22q; 2q, 3q, 7q (associated with lymph node mets); 7p (associated with distant organ mets);**	**3p, 4q, 13q, Ypq,**
**OSCC**	Shi	2011	35/ 44K aCGH, RTPCR	N	**3q, 11q, 19pq, 20pq, 22q**	**4pq, 13q, 18pq, Y**	**3q** *(EIF2B5*, *DVL3*, *AP2M1*, *ABCF3*, *ALG3*, *ECE2*, *CAMK2N2*, *PSMD2*, *EIF4G1*, *FAM131A*, *CLCN2*, *POLR2H*, *THPO*, *CHRD)*, **8q21.11** (*JPH1*, *GDAP1*, *PI15*, *CRISPLD1)*,**8q** (*MYC)*, **11q** (*FGF3)*, **12q** (*FRS2*, *CCT2*, *LRRC10*, *BEST3*, *RAB3IP*, *CNOT2*, *KCNMB4*, *PTPRB PTPRR*, *TSPAN8*, *LGR5*, *CCDC131*, *THAP2*, *TMEM19*, *RAB21)*, **18q** (*C18orf17)*, **19q** (*ZNF507*, *DPY19L3*, *PDCD5*, *ANKRD27*, *RGS9BP*, *NUDT19*, *TDRD12*, *SLC7A9*)	**4q** (*ODZ3)*, **9p**(*CDKN2A*, *CDKN2B)*
**OSCC**	Pack	1999	17/ CGH metaphase spread	N	**3q, 8q, 9q, 12q, 16p, 17, 19, 20q, 22.**	**2q, 3p, 13q, Xq, 4, 5q,18q, 9p, 6q, 12q, 14q, 11q, 1p**	**5p, 11q,**	**1p, 3p, 5q, 6q,11q, 12q**
**OSCC**	Tada	2000	36/ CGH metaphase spread	N	**3q, 2p, 5p, 8q, 7q, 11q, 12p 20q, 20p.**	**3p,4q, 5q, 9p, 13q, 11q, 18q,**	**2q, 7q, 11q, 20q.**	
**HNSCC**	Jung	2010	231 total; 91 for 4.4K aCGH	Y	n/a	**9p (more common in HPV negative). 16q (more common in HPV positive)*(****APP-BP*, *DDX28*, *ZDHHC1*, *SLC9A5*, *NUTF2*, *CIRH1A*, *NOB1*, *FOXC2*, *WDR59*, *OSGIN1*, *COX4NB*, *APRT)*	**11q, 13p (more common in HPV negative).**	n/a
**OPSCC**	Klussmann	2009	60/ CGH metaphase spread	Y (28 HPV16 pos)	**3q** *(ATR*, *PIK3CA*, *TP63*, *TERC*, *DCUN1D1*, *LAMP3*, *RPL35A)*, **8q, 17q** (overall), **Xp** (more in HPV pos)	**3p***(FHIT)*, **4q, 9p** *(CDKN2A)*, **13q** (overall); **3p, 5q, 9p, 15q, and 18q** (more in HPV neg); **16q** (more in HPV pos)	**11q**(more in HPV neg) *(CCND1*, *CTTN*, *FADD)*	
**VSCC**	Yangling	2007	unavailable	Y	**3q,12q** (more common in HPV pos) **8q** (more common in HPV neg)	Unavailable	unavailable	unavailable
**VSCC**	Purdie	2010	6 / 25k nsp SNP array	Y (All HPV positive)	**1p, 1q, 8p, 8q, 9p, 9q, 19, 20p, 20q, 22q, Xp, Xq**	**n/a**	**n/a**	**2q, 3p, 10p, 10q, 14q, Xp**
**VSCC CSCC**	Huang	2005	28 CSCC, 8 VSCC,7 CANCER CELL LINES	Y (25/28 CSCC, 6/8 VSCC)	**2p and q, 5q, 6p and q, 8p, 9p, 10p, 11p and q, 12p and q, 13q, 15q, 18q, 19q, 22q** (CSCC); **1p and q, 3q, 5p, 8q, 14q, 20p, X** (CSCC and VSCC),	**2q, 3q, 6q, 7p, 10p, 12p, 19p** *(CDKN2A)* **and q, 20q** (CSCC); **3p** *(FHIT*, *FRA3B*, *MLH1)*, **4p and q, 5q, 7q, 8p, 10q, 11p** *(WT1*, *WT2*, *CDKN1C)* **and q,16q, 17p and q,22q** (CSCC and VSCC); **18q** (VSCC)	**3q (commonly found in HPV 16 pos)***(PIK3CA*, *hTR*, *ETS1*, *ETV5*, *OPA1)*, **8q commonly found in HPV 18 pos)** *(c-MYC*, *eIF3*, *PSCA)*, **11q, 19q** (CSCC and VSCC), **1q, 5p, 6p, 9p, 17q, 20, Xq** (CSCC)	
**CSCC**	Policht	2010	19/FISH	N	**1pq, 2pq, 3pq, 4p, 5p, 6pq, 7p, 8q, 11pq, 20q, Xpq.**	**1q, 2q, 3p, 7p, Xpq,**	**n/a**	n/a
**CSCC**	Rao	2004	72/ CGH metaphase spread	Y (all high risk HPV pos)	**1pq, 3q** *(PIK3CA*, *eIF-5A2*, *CCNL1***), 5p, 8q, 9q, 13q, 19p 20pq, Xq.**	**2q, 3p, 4pq, 11q, 13q, 17p.**	**1p, 2q, 7q, 8q** *(PRL-3*, *WISP)*, **9p, 10q, 11q** *(CCND1/PRAD1)*, **11q21, 12q, 14q, 17pq, 18p, 19q.**	**2q** (*CFLAR*, *CASP10* and *PPP1R7*), **11q, 13q.**
**CSCC VSCC**	Thomas (meta analysis)	2003	293 (CSCC), 30 (VSCC)	Y (all VSCC HPV pos)	*3q (CSCC and VSCC) esp HPV16 pos cscc	*3p, *11q (CSCC and VSCC)	n/a	n/a
**PSCC**	Alves	2001	21/ CGH metaphase spread	N	*(5p, 8q, 16p, 19q, 20q, 22q).	**1–14pq, 15q, 16q, 17–19pq; 21q, 20p.**	**12q** *(MDM2)*	*4q, 13q.
**PSCC**	**current study**		64/aCGH	Y	^**+**^**1p and ^1q,** ^**+¥**^**2p,** ^**+**^**3p** (*PRKCI*, *PIK3CA*, *DCUN1D1*, *LAMP3*, *RPL35A)*, **4q, 5p** *(SKP2*), ^**+**^**6p,** ^**+¥**^**7p and 7q,** ^**+**^**8p** *(MYC)*, ^**+**^**9p,** ^**¥**^**10q, 11p, 12q, 14q, 15q, 16p, 17q, 19pq,** ^**+**^**20pq, 21q, 23q, 22q.**	**1p, 2 p and** ^**+**^**q** *(PLCD4)*, **3p** (*RBMS3*, *PLCD1*, *CACNA2D3)*, **and 3q, 4pq, 5q, 6pq, 7q, 8p, 9pq, 10pq,** ^**¥**^**11p and 11q** *(CPT1A*, *CCND1*, *FGF4/FGF3*, *PPP2R1B)*, **12pq, 13q, 14q, 15q, 16q, 17pq, 18pq, 19p, 20pq, 21q, 22q, 23q, 24q**	**1q, 3q** *(PIK3CA*, *SENP5*, *CLDN1*, *TNK2*, *FBXO45*, *FNDC3B*, *ACTL6A/BAF53****)*, 5p, 8q, 11p (***CPT1A*, *FGF3/4 and CCND1)*.	n/a

Comparison of copy number gains, losses, amplifications and deletions between penile, oesophageal, head and neck, vulval and cervical squamous cell carcinomas

Within other gained (5p15.33-p11) and amplified (5p15.33-p11) lies SKP2. It comprises the substrate recognition unit of the SCF^SKP2^ ubiquitin ligase complex and has been involved in the ubiquitin driven breakdown of cyclin dependent kinase inhibitor p27 (a dosage dependent tumor suppressor protein), which is an important regulator of G1 progression. Increased levels of expression have been demonstrated in squamous and non squamous cell carcinomas and it has been linked with tumor progression and poor prognosis [34–37]. Apart from PIK3CA, amplification of 3q27.2-q29 also encompasses SENP5, CLDN1 and TNK2. SENP5 is a SUMO (small ubiquitin-like modifier) specific protease. The addition of SUMO proteins is responsible for the reversible post translational modification of proteins. SUMOS are conjugated to cellular regulators, which changes the localization, activity and stability of the substrates. SENP5 plays an important role in many cancer types, including esophageal squamous cell carcinoma [38]. CLDN1 is a member of a family of principal proteins important for tight junction formation and function. CLDN1 has been detected at significantly increased levels in cervical cancer, compared to normal cervical epithelium, and has been implicated in cervical cancer progression. TNK2 is a ubiquitously expressed non-receptor tyrosine-protein and serine/threonine-protein kinase that is implicated in cell spreading and migration, cell survival, cell growth and proliferation. TNK2 forms a robust complex with all isoforms of AKT and directly regulates AKT's plasma membrane recruitment, thereby bypassing the PI3-kinase pathway. This process is facilitated by a novel phosphorylation event in AKT, Tyrosine 176 that is invariant from yeast to mammals. TNK2 gains have been found in breast, prostate, lung, pancreas, brain, Ewing’s sarcoma and the pharmaceutical agent dasatinib is a known inhibitor [39].

FBXO45 binds to TP73, a pro-apoptotic gene, and initiates its proteosome dependent degradation [40]. FNDC3B is contained in the amplified region 3q26.1-q27.2, inducing the epithelial-to-mesenchymal transition (EMT) and activating several cancer pathways, including PI3-kinase/Akt, RB1 and TGFβ signaling [41]. CPT1A is an isoform of carnitine palmitoyl transferase 1 that has been demonstrated to interact with BCL2 [42]. Some of these potential driver genes exist as part of molecular pathways for which therapeutic agents are either already in use or are in various stages of clinical trials.

Focusing on our HPV subgroup, we showed gain of 1q to be more frequent in HPV-positive cases and gain of 2p, 7p and 10q, as well as loss of 11p to be more frequent in HPV-negative cases. Only a subset of the data on other squamous cell carcinomas includes HPV DNA analysis, but not all findings are in concordance with those of PSCC. For example, Yangling *et al*. found gain of 3q and 12q to be more common in HPV-positive VSCC cases and gain of 8q more common in HPV-negative [43]. Differences were also seen in comparison of HPV cohorts in oropharyngeal squamous cell carcinomas [18, 44], which highlighted Xp gain and 16q loss more common in HPV-positive cases, and 3p, 5q, 9p, 15q and 18q loss more common in HPV-negative cases [18]. In contrast to these studies, a higher frequency of 1q gain in our HPV-positive cohort is in accordance with HPV-positive VSCCs [45] and CSCCs [46–48]. Further analysis by Jung *et al*. also demonstrated a distinction between HPV-positive (DNA/RNA positive) and HPV-negative (DNA/RNA negative and DNA-positive/RNA-negative) tumors, with clustering of the HPV-positive group, which was not reflected in our PSCC data.

Although there is lack of gross correlation between genetic copy number change and HPV status, more detailed analysis of PSCCs may reveal more striking differences between the HPV groups, as disclosed by next-generation sequencing in patients with head and neck squamous cell carcinoma that showed more mutations in HPV-negative than HPV-positive tumors [49], as well as recent analysis of esophageal epithelial cell lines *in vitro* suggests that HPV infectivity was independent of inflammation and may play a less significant role than first thought [14].

In addition, lymph node metastases were associated with chromosomal amplifications in OSCC at 2q12–14, 3q24–26, and 7q21–31, while distant metastasis correlated to amplification at 7p13–21 [50]. This is in contrast to our data that highlighted higher frequency of statistically significant losses in lymph node negative PSCCs. As lymph node status is a marker of prognosis in PSCC, given further analysis, these genetic aberrations may have implications with regard to development of early molecular prognostic indicators. The statistically significant, small genetic differences in high stage PSCC also highlight potential targets for further analysis of PSCC progression.

The differences in genomic profile between PSCCs and other HPV related squamous cell carcinomas may, in part, be attributed to intrinsic genetic differences, the different methods of CGH used or the number of cases analyzed.

## Conclusion

It has long been suggested that SCCs in different locations may develop through similar molecular pathways and that HPV and non-HPV pathways are responsible in the pathogenesis of PSCC. The correlations between the clinico-pathological data and copy number aberrations in our study supports this, but with the differences in genetic aberrations between PSCCs and other types of squamous cell carcinomas this remains unproven. Nonetheless, the correlations demonstrated here could play an important role in PSCC natural history and progression and highlight potential driver genes, which may feature in molecular pathways for existing therapeutic agents.

Further analysis of PSCCs would need to be conducted to establish whether or not there is a final common pathway of HPV and non-HPV associated PSCCs and this would need to include next generation sequencing, cell culture studies, acquisition of paired genetic information from metastatic lymph node tissue and correlation with survival data.

## Supporting Information

S1 Table Penile Cancer Overall Recurrent Copy Number Gains and Losses. (DOCX)

S2 Table Clinicopathological Subgroup Analysis of Differential Copy Number Changes. (DOCX)
