# Supplementary material for: DNA Copy Number Aberrations, and Human Papillomavirus Status in Penile Carcinoma. Clinico-Pathological Correlations and Potential Driver Genes
Source: PLoS One. 2016 Feb 22;11(2):e0146740. doi: 10.1371/journal.pone.0146740 (PMC4763861; doi:10.1371/journal.pone.0146740)
Supplement: S1 Table — (DOCX) [file pone.0146740.s001.docx]

**S1 TABLE: Penile Cancer Overall Recurrent Copy Number Gains and Losses**

| **Chromosome** | **Gene locus** | **Frequency** |
| --- | --- | --- |
| 1 | p13.3-q44 | 88% |
| 2 | p21-q33.3 | 83% |
| 3 | p12.3-q29 | 86% |
| 4 | q34.2-q35.2 | 39% |
| 5 | p15.33-p11 | 67% |
| 6 | p12.3-q12 | 44% |
| 7 | p22.3-q36.3 | 88% |
| 8 | p12-q24.3 | 84% |
| 9 | p24.3-q34.3 | 88% |
| 11 | p12.3-q14.1 | 39% |
| 12 | q24.11-q24.33 | 42% |
| 14 | q11.2-q32.33 | 66% |
| 15 | q11.2-q24.3 | 47% |
| 16 | p11.2-q24.3 | 77% |
| 17 | q21.33-q25.3 | 53% |
| 19 | p13.11-q13.43 | 64% |
| 20 | p13-q13.33 | 84% |
| 21 | q11.1-q22.3 | 66% |
| 23 | q21.31-q28 | 44% |

**GAINS**

**LOSSES**

| **Chromosome** | **Gene locus** | **Frequency** |
| --- | --- | --- |
| 1 | p36.33-p34.2 | 56% |
| 1 | p32.3-p32.2 | 47% |
| 2 | q33-q37.3 | 86% |
| 2 | p22.3-p16.3 | 55% |
| 2 | p24.2-23.2 | 40% |
| 3 | p26.3-q11.1 | 83% |
| 3 | q13.13-13.31 | 40% |
| 4 | p16.3-p11 | 55% |
| 4 | q28.3-q35.2 | 55% |
| 4 | q13.3-q28.3 | 47% |
| 5 | q35.1-q35.2 | 64% |
| 5 | q33.3-q35.1 | 48% |
| 5 | q13.2 | 44% |
| 6 | q16.3-q25.2 | 67% |
| 6 | p21.1-p12.3 | 42% |
| 7 | q31.1-q36.3 | 64% |
| 8 | p23.3-q11.1 | 72% |
| 9 | p23-p21.3 | 53% |
| 9 | q33.3-q34.3 | 52% |
| 10 | q23.1-26.3 | 77% |
| 10 | p15.3-p11.1 | 66% |
| 10 | p21.3-q23.1 | 64% |
| 11 | q12.2-q25 | 81% |
| 11 | p15.5-p14.3 | 64% |
| 12 | p13.3-p11.21 | 61% |
| 12 | q24.23-q24.31 | 39% |
| 13 | q12.2-q31.1 | 55% |
| 13 | q31.1-q34 | 47% |
| 14 | q23.3-q24.3 | 45% |
| 15 | q21.1-q25.3 | 56% |
| 15 | q25.3-q26.3 | 55% |
| 16 | q22.2-q24.3 | 64% |
| 16 | q12.1-q21 | 39% |
| 17 | p13.3-p11.1 | 69% |
| 17 | q25.1-q25.3 | 55% |
| 17 | q12-q21.33 | 48% |
| 18 | q12.1-q23 | 75% |
| 18 | p11.31-q12.1 | 55% |
| 19 | p13.3-p12 | 58% |
| 20 | p13-p11.23 | 52% |
| 20 | q12-q13.2 | 39% |
| 21 | q21.2-q22.3 | 69% |
| 22 | q11.1-q13.3 | 66% |
| 23 | q28 | 45% |

**AMPLIFICATIONS**

| **Chromosome** | **Gene locus** | **Frequency** |
| --- | --- | --- |
| 1 | q32.2 | 6% |
| 3 | q27.2-q29 | 11% |
| 3 | q26.1-q27.2 | 8% |
| 5 | p15.2-p14.3 | 9% |
| 5 | p14.3-p11 | 8% |
| 5 | p15.33-p15.2 | 6% |
| 8 | q21.13-q24.3 | 23% |
| 11 | p14.1-p12 | 6% |
