# Supplementary material for: DNA Copy Number Aberrations, and Human Papillomavirus Status in Penile Carcinoma. Clinico-Pathological Correlations and Potential Driver Genes
Source: PLoS One. 2016 Feb 22;11(2):e0146740. doi: 10.1371/journal.pone.0146740 (PMC4763861; doi:10.1371/journal.pone.0146740)
Supplement: S2 Table — (DOCX) [file pone.0146740.s002.docx]

**S2 TABLE: Clinico-pathological Subgroup Analysis of Differential Copy Number Changes**

| **Subgroup** | **Chromosome** | **Differential Gain** | **Differential Loss** | **Differential Amplification** |
| --- | --- | --- | --- | --- |
| **Basaloid** | 1q | 153,406,844-153,890,950, 168,360211-168,582,272, 169,081,525-169,575,212 170,011,827-170,919965 (p<0.05) |  |  |
|  | 3q | 193,646,036-193,914,131  (p=0.04, region close to telomere); |  |  |
|  | 11p and 11q |  | 26,348,389 - 26,748,623, 87,095,202 - 88,351,544, 129,739,104 - 129,887,737 130,767,849 -134, 927, 296  (close to telomere) (p<0.05) |  |
|  | 13q |  | 88,676,494 - 92,029,220 95,059,210 - 95,931,996  (p=0.03) |  |
|  | 15q |  | 31,951,325-32,376,877 (p=0.03); |  |
|  | 16q |  | 57,823,567 - 57,992,604 (p=0.02),  58,540,478 - 59,005,775 (p<0.01)  81,387,855 - 81,836,485 (p=0.03); |  |
|  | 3q |  |  | 121,495,658 - 121,972,355 167,516,772 - 169,326,472 (p<0.05); |
|  | 8q |  |  | 125,100,708 - 127,884,025 129,247,390 - 129,501,508 (p<0.05). |
| **Non verrucous** | 11p |  | 47,518,955-47,867,829 (p<0.05) |  |
|  | 17q |  | 46,618,787 - 47,536,003, 47,581,243 - 477,729,834 48,652,564 - 49,397,628 |  |
| **Usual** | 3p |  | 16,287394 - 16,435,851, 16,455,294 - 16,785,131 72,520,499 - 72,993,504 (p<0.05). |  |
| **Grade 1+2** | 2p |  | 18,441,084 - 18,611,588, 36,236,583 - 36,349,058, 71,974,149(p<0.05) |  |
|  | 5p and q |  | 39,312,375 - 39,608,343, 125,596,256 - 125,957,381(p<0.05) |  |
| **Grade 3** | 8q |  | 98,217,510 - 98,381,393, 98,622,574 - 99,325,883 99,650,748(p<0.05) |  |
|  | 11p |  | 47,518,955 - 47,671(p<0.05) |  |
| **Stage 3 and 4** | 5q |  | 142,012,666 - 142,175,698 (p=0.03), |  |
|  | 6p |  | 45670490 - 45,851,371  (p<0.01) |  |
|  | 18p |  | 125,640,882 - 126,607,828, 128,096,371 - 128,625,942 (p<0.05). |  |
|  |  |  |  |  |
| **Node negative** | 2p |  | 36,920,270 - 37,578,954 38,621,890 - 40,066,256 |  |
|  | 4q |  | 101,026,920 -102,065,074 (p=0.03); |  |
|  | 9q |  | 91,181,836 - 91,566,892 (p<0.05) |  |
|  | 10q |  | 114,067,767 - 115,375,350 (p<0.05); |  |
|  | 15q |  | 50,550,602 - 52,277,893 (p<0.05); |  |
|  | 20p |  | 8,412,274 - 8,758,526,  8,819,804 - 9,531,260,  17,184,216 - 17,484,732, 17,852,916 - 19,328,441 (p<0.05). |  |
